# *Ganoderma lucidum* Polysaccharide Seed Dressing Induces Systemic Resistance Against Fusarium Head Blight and Sharp Eyespot in Wheat

**DOI:** 10.3390/jof12070538

**Published:** 2026-07-22

**Authors:** Yao Zhu, Ping He, Wanxiu Zhang, Xiang He, Xinli Li, Xiaolong He, Xiaopeng Gao, Baotong Wang, Jianzhao Qi, Yueqin Liu, Pengfei Jin

**Affiliations:** 1 Shaanxi Key Laboratory of Research and Utilization of Resource Plants on the Loess Plateau, Engineering Research Center of Microbial Resources Development and Green Recycling of the University of Shaanxi Province, Research and Development Centre of Ecological and Sustainable Application of Microbial Industry of the Loess Plateau in Shaanxi Province, College of Life Science, College of Yan’an Medical, Yan’an University, Yan’an 716000, China; 2 State Key Laboratory for Crop Stress Resistance and High-Efficiency Production, College of Plant Protection, Northwest A&F University, Yangling 712100, China; 3 Center of Edible Fungi, Northwest A&F University, Yangling 712100, China

**Keywords:** *Ganoderma lucidum* polysaccharide, wheat, Fusarium head blight, sharp eyespot, control effect

## Abstract

*Ganoderma lucidum* polysaccharide (GLP) exhibits prominent antibacterial and antioxidant activities. This study evaluated the effects of GLP seed soaking at two concentrations (4 g/100 kg, GLP4; 8 g/100 kg, GLP8) on wheat growth promotion and induced resistance against wheat sharp eyespot caused by *Rhizoctonia solani* and Fusarium head blight (FHB) mainly caused by *Fusarium graminearum*. Physiological and agronomic analyses showed that GLP treatment increased the germination rate of all tested wheat cultivars (moderately resistant: Xiaoyan 22, Sumai 3; moderately susceptible: Mingxian 169, Xinong 873) by over 2%. Moderately susceptible and resistant cultivars presented average plant height increases of 0.5 cm and 2 cm, respectively, with most cultivars showing a height increase of approximately 3 cm. GLP significantly elevated leaf chlorophyll content by over 5% and differentially regulated malondialdehyde (MDA) levels: MDA decreased by 34–68% in Sumai 3 and Mingxian 169 but increased by 11–23% in Xiaoyan 22. Pot assays verified that GLP yielded over 10% control efficacy against both diseases. Overall, GLP seed soaking effectively promotes wheat growth, activates defense responses, and enhances host resistance to *R. solani* and *F. graminearum* infections.

## 1. Introduction

Wheat is one of the staple food crops in China and plays an irreplaceable role in national food security [1]. In recent years, the incidence and damage of wheat fungal diseases have generally increased, severely restricting high and stable yields as well as grain quality safety of wheat [2,3]. Among these diseases, wheat sharp eyespot caused by *Rhizoctonia solani* and Fusarium head blight (FHB) predominantly incited by *Fusarium graminearum* are typical soil-borne basal stem disease and air-borne spike disease, respectively. Both diseases prevail in major wheat-producing regions worldwide and cause severe losses, including plant lodging, reduced 1000-kernel weight, and mycotoxin accumulation in grains, thereby exerting markedly adverse impacts on wheat yield formation and grain quality safety [4,5,6]. At present, chemical fungicides constitute the primary measure to control wheat sharp eyespot and FHB. Nevertheless, pathogens rapidly mutate and generate novel virulent pathotypes, which frequently lead to fast breakdown of major wheat resistance genes. Moreover, long-term and excessive application of agrochemicals inevitably triggers severe environmental pollution [7,8,9,10]. Against this backdrop, exploring eco-friendly and sustainable alternative strategies for disease management has become a key research topic in plant protection. Plant immune elicitors are functional substances capable of activating endogenous plant immune systems and triggering defensive responses in host plants. Featuring favorable environmental compatibility and low risks of pathogen resistance development, these compounds contribute to constructing eco-friendly disease management systems and bear great strategic significance for guaranteeing safe wheat production [11,12].

Edible mushroom polysaccharides possess multiple pharmacological activities, including anti-tumor, antioxidant, immunomodulatory, hypoglycemic, hypolipidemic, anti-viral, anti-inflammatory, and hepatoprotective effects [13,14,15,16,17,18,19]. Accumulating evidence has demonstrated that *Ganoderma lucidum* polysaccharide (GLP) effectively activates plant molecular immune systems, enhances stress and disease resistance of plants, and alleviates the severity of fungal diseases [20,21,22]. Seed coating with GLP facilitates wheat seed germination and seedling growth; meanwhile, GLP treatment induces significant alterations in leaf defense enzyme activities, chlorophyll contents, and malondialdehyde (MDA) levels in wheat seedlings [21,23]. Fu et al. reported that GLP confers tomato resistance against tomato yellow leaf curl virus while promoting seed germination and seedling development of tomato [24,25]. In addition, Gong et al. indicated that functional carbohydrates such as chitosan oligosaccharide and lentinan can modulate crop growth, activate plant defensive responses, and strengthen host resistance to viral and fungal diseases [26,27,28,29,30]. Apart from single application, polysaccharides have also been investigated for combined use with chemical fungicides to achieve synergistic effects. Jiang et al. verified that mixtures of lentinan and hexaconazole can control two soil-borne diseases, wheat sharp eyespot and wheat root rot [31]. Such combined formulations reduce fungicide dosage, concurrently suppress multiple diseases, lower agrochemical residues in the environment, and extend the persistent efficacy of fungicides. Li et al. further confirmed that the combination of fluopyram and lentinan exhibits outstanding control efficacy against cucumber downy mildew, cutting the application rate of fluopyram by 25% [32].

In this study, seed coating combined with indoor pot trials was performed using four wheat cultivars to systematically evaluate the control efficacy of GLP against FHB and wheat sharp eyespot. Chlorophyll and MDA contents were measured as physiological biomarkers to dissect the priming resistance mechanism mediated by GLP. Meanwhile, growth-related indicators including germination rate and plant height of each cultivar were determined to comprehensively clarify the regulatory roles of GLP in wheat growth and development. Disease indices were surveyed, and relative control efficiencies were calculated to synthetically assess GLP-induced resistance to the two fungal diseases. As novel biocontrol agents, biopesticides are characterized by low toxicity, low residual levels, and safety to non-target organisms and ecological environments [33,34,35]. They mitigate the resistance crisis induced by chemical pesticides and support high-quality crop production without disrupting farmland ecosystems and ecological balance, thus attracting extensive research attention globally [36,37,38]. The development of GLP extracts as botanical pesticides for green management of wheat fungal diseases not only meets the strategic demands of sustainable agriculture, but also carries important theoretical values and broad application prospects.

## 2. Materials and Methods

### 2.1. Plant and Fungal Material

Moderately susceptible wheat cultivars Mingxian 169 and Xinong 873, together with moderately resistant cultivars Sumai 3 and Xiaoyan 22, were adopted in this study. All wheat seeds were provided by the Laboratory for Wheat Pathogenic Fungal Monitoring and Disease Resistance Genetics, Northwest A&F University.

Test strains of *F. graminearum* and *R. solani* were preserved and supplied by the Microbiology Laboratory, College of Life Sciences, Yan’an University. Both fungal strains were maintained on PDA slants at 4 °C. Prior to use, the strains were transferred onto fresh PDA plates and incubated at a constant temperature of 25 °C for activation.

Wild-type *G. lucidum* strain G-1 was isolated from wild fruiting bodies collected in Mohe City, Daxing’an Mountains, Heilongjiang Province, China. Molecular identification was performed via internal transcribed spacer (ITS) sequence analysis, with its GenBank accession number JQ781853. For strain activation, preserved cultures were inoculated onto potato dextrose agar (PDA) plates and incubated in darkness at 25 °C for 7 days. For liquid fermentation, mycelial plugs from activated plates were inoculated into liquid medium, statically cultured at 25 °C for 2 days, followed by shaking incubation at 180 rpm for another 8 days. The optimal growth temperature for mycelia of this strain was 25 °C, and mycelial growth was markedly inhibited when temperatures exceeded 25 °C.

### 2.2. Preparation of G. lucidum Polysaccharides

**Figure 1:**
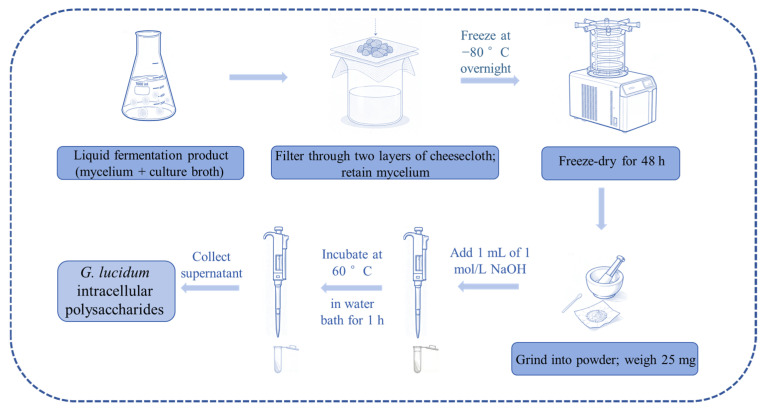
Schematic diagram of intracellular polysaccharide extraction from *G. lucidum* mycelia obtained by liquid fermentation.

Intracellular polysaccharides were extracted from *G. lucidum* mycelia via water extraction and ethanol precipitation. Mycelia obtained from liquid fermentation were harvested by double-layer gauze filtration, freeze-dried for 48 h, and pulverized for subsequent use. Briefly, 25 mg of ground mycelial powder was mixed with purified water at a solid-liquid ratio of 1:3, followed by water-bath extraction at 90 °C for 2 h. The supernatant was collected after centrifugation, and the residue was re-extracted twice; all supernatants were pooled together. The combined supernatant was inactivated in a water bath at 60 °C for 1 h, mixed with 1 mL of 1 mol/L NaOH, and supplemented with absolute ethanol to achieve a final ethanol concentration of 75%. The mixture was incubated at 4 °C overnight for polysaccharide precipitation. The precipitate was harvested by centrifugation, redissolved in purified water, and subjected to repeated ethanol precipitation. This purification process was repeated 2–3 times, and the final precipitate was freeze-dried to obtain intracellular *G. lucidum* polysaccharide. Figure 1 shows the flow chart for the extraction of intracellular polysaccharides from *G. lucidum* mycelia using the water extraction and ethanol precipitation method.

Determination of polysaccharide content: Briefly, 1 mL of the sample solution was mixed with 1 mL of 5% phenol solution and 5 mL of concentrated sulfuric acid. After thorough mixing, the mixture was brought to a constant volume with distilled water and incubated at room temperature for 30 min before absorbance measurement. The polysaccharide content was calculated according to the standard curve.

### 2.3. Wheat Germination and Emergence Test

Seeds were treated by mixing *G. lucidum* polysaccharides at doses of 4 g/100 kg and 8 g/100 kg wheat seeds, with a control group treated with clean water. Germination test: Seeds of uniform plumpness and size were selected from each moderately susceptible and moderately resistant variety. The four varieties were allocated to three treatment groups and germinated at 25 °C room temperature. The germination rates of wheat seeds in each treatment were recorded and calculated. Calculation formula: Germination rate (%) = (Number of germinated seeds in each treatment on day 6/Total number of seeds tested) × 100. Emergence trial: Following the method of Dang et al. with minor modifications, sow 10 seeds per treatment in two replicates within pots [39]. Cultivate the four varieties under two disease conditions in the greenhouse of the College of Life Sciences, Yan’an University. Record growth status at 7, 14, and 21 days post-emergence and measure plant height across treatments.

### 2.4. Indoor Potted Plant Disease Resistance Trial Using G. lucidum Polysaccharide Seed Treatment

The pot experiment was carried out in an artificial climate greenhouse. Plastic pots with an inner diameter of 16 cm were filled with 1 kg of nutrient substrate (purchased from Hengxian Seedling Substrate Factory, Shouguang City, Shandong Province, China), and the substrate water content was adjusted to 60%. Each pot was irrigated with 20 mL of single-pathogen inoculum, with separate pots inoculated with either *R. solani* suspension (OD_420_ = 0.56) or *F. graminearum* spore suspension (1 × 10^5^ spores/mL). The pots were sealed and incubated in darkness for 24 h to allow uniform distribution of pathogens in the substrate. Three seed coating treatments were established: CK (seed coating with distilled water), GLP4 (4 g GLP per 100 kg seeds), and GLP8 (8 g GLP per 100 kg seeds). The tested wheat cultivars included moderately resistant lines Xiaoyan 22 and Sumai 3, as well as moderately susceptible lines Mingxian 169 and Xinong 873. Ten plump and uniform wheat seeds were sown in each pot, with two biological replicates for each cultivar × treatment combination, and blank substrate pots were set as additional controls. The greenhouse was maintained at a constant temperature of 25 ± 1 °C under a 14 h light/10 h dark photoperiod, a photosynthetic photon flux density of 300 μmol·m^−2^·s^−1^, and relative humidity of 65–70%, with daily environmental monitoring and adjustment. No chemical fertilizers or pesticides were applied throughout the whole growth period. The pots were weighed and supplemented with water every 2 days to sustain substrate moisture at 55–60%, and water was poured slowly along pot edges to avoid water-logging or drought stress. Disease indices of FHB and wheat sharp eyespot were investigated at 7 d, 14 d, and 21 d after seedling emergence. All plants in each treatment were surveyed with two replicates, and relative control efficacy was calculated according to Formulas (1) and (2) described in the main text. Disease severity of both seedling diseases was assessed using a unified 0–5 rating scale: Grade 0, no lesions on stem base, plants fully healthy; Grade 1, scattered light brown lesions covering <10% of stem base for FHB, punctate brown lesions encircling less than 1/4 of stem circumference for sharp eyespot; Grade 2, FHB lesions covering 10–30% without seedling wilting, sharp eyespot lesions encircling 1/4–1/2 of stem circumference with unobvious retarded growth; Grade 3, FHB lesions covering 30–50% accompanied by slight leaf chlorosis, sharp eyespot lesions encircling 1/2–3/4 of stem circumference with weakened plant vigor; Grade 4, FHB lesions covering >50% with severe seedling chlorosis and wilting, sharp eyespot lesions fully encircling stem base with obvious plant wilting; Grade 5, complete stem base rot and whole plant death for FHB, rotted and broken stem base leading to seedling mortality for sharp eyespot.

Disease Index = ∑(Number of diseased plants at each stage × Relative stage value)/(Total number of plants surveyed × Maximum stage value for each disease) × 100(1)

Control Effectiveness = (Disease Index of Untreated Control Area − Disease Index of Treated Area)/Disease Index of Untreated Control Area × 100%(2)

### 2.5. Determination of the Effect of G. lucidum Polysaccharide Seed Treatment on Chlorophyll Content in Wheat

Referring to the method described by Song et al. [40], the contents of chlorophyll a and chlorophyll b in wheat leaves were determined at 7, 14 and 21 days after seed coating with different concentrations of GLP. Briefly, 0.2 g fresh leaf tissue was weighed and immersed in 10 mL of 95% ethanol for 48 h in darkness at 4 °C. The supernatant was collected, and its absorbance was measured at wavelengths of 665 nm and 649 nm using a UV-visible spectrophotometer (YOKE, Shanghai, China), with three replicate readings for each sample. The formulas for calculating chlorophyll concentrations are shown as follows:

Ca = 13.95 OD_665-_6.88 OD_649_ (3)

Cb = 24.96 OD_649-_7.32 OD_665_ (4)

Chlorophyll content (mg/g FW) = C _Chl_ × V _extraction_ × dilution factor/(sample fresh weight × 1000) (5)

### 2.6. Determination of the Effect of G. lucidum Polysaccharide Seed Treatment on Malondialdehyde Content in Wheat

Following the method of Krishankumar et al. with minor modifications, MDA content (μmol/g) was determined using the thiobarbituric acid assay [41]. The calculation formula is:

MDA (μmol/g FW) = [6.425 × (A_532_ − A_600_) − 0.559 × A_450_] × Vt/(Vs × FW) (6)

where: FW—Fresh weight of sample (g); Vt—Total volume of extract (mL); Vs—Volume of extract used for measurement (mL).

### 2.7. Statistical Analysis

Data are mean ± standard error with three replicates per treatment (*n* = 3). One-way ANOVA was used to compare GLP treatments within each wheat variety, followed by Duncan’s multiple range test (*p* < 0.05). Pearson correlation was applied to assess relationships between physiological traits and disease index. Normality and homogeneity were checked by Shapiro–Wilk and Levene’s tests. SPSS26.0 was used for statistics and OriginPro2023 for graphing.

## 3. Results

### 3.1. Determination of G. lucidum Polysaccharide Content

The polysaccharide content of *G. lucidum* mycelia was determined by the phenol-sulfuric acid method, with a measured polysaccharide concentration of 0.98 ± 0.06 mg/mL.

### 3.2. Effects on Wheat Seed Germination Rate

Germination of wheat seeds treated with *G. lucidum* polysaccharides induces systemic disease resistance and enhances seed germination rates. As shown in Table 1, both moderately resistant and moderately susceptible wheat varieties exhibited higher germination rates than the control group following polysaccharide seed dressing. At a dosage of 8 g per 100 kg of wheat seeds, the germination rates of moderately resistant wheat varieties Xiaoyan 22 and Sumai 3, and moderately susceptible varieties Mingxian 169 and Xinong 873, increased by 3.8%, 6.4%, 19.5%, and 2.6%, respectively, compared to the control group. This demonstrates that *G. lucidum* polysaccharides exert a more pronounced stimulatory effect on seed germination in the moderately susceptible wheat variety Mingxian 169.

### 3.3. Impacts on Wheat Seedling Development

#### 3.3.1. The Way Fusarium Head Blight Affects the Growth of Wheat Seedlings

*G. lucidum* polysaccharide seed treatment was used in this experiment. At 7, 14, and 21 days following full emergence, the moderately resistant cultivars Xiaoyan 22 and Sumai 3 showed noticeably higher plant heights than the control group, with growth reaching 2 cm above the control, according to Table 2. The 4 g/100 kg seed treatment with *G. lucidum* polysaccharides showed a more noticeable growth-promoting impact for the moderately susceptible cultivars Mingxian 169 and Xinong 873, surpassing the control group by more than 0.5 cm. The *G. lucidum* polysaccharide 8 g/100 kg seed treatment group’s Xiaoyan 22 and Sumai 3 seed heights were more than 7% higher than those of the control group 21 days after full emergence. This suggests that *G. lucidum* polysaccharide promotes early growth in these moderately resistant wheat cultivars more strongly as its concentration rises. The Mingxian 169 and Xinong 873 cultivars in the *G. lucidum* polysaccharide 4 g/100 kg seed treatment group showed more than 2% more growth than the control group 21 days after full emergence. This reveals that the 4 g/100 kg seed treatment group exhibits a greater promotion effect on the early development of moderately susceptible wheat types.

#### 3.3.2. Sharp Eyespot’s Effects on Wheat Seedling Growth

In the wheat types Xiaoyan 22, Sumai 3 (resistant), and Mingxian 169 (moderately sensitive), this experiment showed that seed treatment with *G. lucidum* polysaccharides stimulated growth; growth increased gradually as polysaccharide content increased (see Table 3). The somewhat resistant wheat cultivars showed the fastest growth among them. The moderately sensitive variety Mingxian 169 and the moderately resistant variants Xiaoyan 22 and Sumai 3 outgrew the control group by more than 2 cm seven days after full emergence. It is shown that *G. lucidum* polysaccharides can promote the healthy growth of wheat plants while suppressing wheat sharp eyespot in the moderately sensitive variety Mingxian 169 and the moderately resistant variants Xiaoyan 22 and Sumai 3.

### 3.4. Indoor Potted Control Efficacy of G. lucidum Polysaccharide Seed Dressing Against Wheat Fusarium Head Blight and Wheat Sharp Eyespot

#### 3.4.1. The Extent to Which Indoor Potted Plants Prevent Fusarium Head Rot in Wheat

Following seed treatment with *G. lucidum* polysaccharides at varying doses, it was observed that after 7, 14, and 21 days of treatment with an 8 g/100 kg seed dose, the control efficacy against FHB in the moderately resistant wheat variety Sumai 3 exceeded 50%, while efficacy against the moderately susceptible variety Xinong 873 exceeded 30%. Observation of Table 4 indicates that seed treatment with *G. lucidum* polysaccharides at both 4 g/100 kg and 8 g/100 kg seed doses demonstrated certain control efficacy for both moderately resistant and moderately susceptible wheat varieties, with the highest efficacy observed for Sumai 3.

#### 3.4.2. Indoor Potted Control Efficacy of *G. lucidum* Polysaccharide Seed Dressing Against Wheat Sharp Eyespot

All treatments showed differing degrees of effectiveness against wheat sharp eyespot after seed treatment with *G. lucidum* polysaccharides at various doses. Of these, the Xinong 873 variety showed the best disease control (Table 5). The disease control efficiency against wheat sharp eyespot in Xiaoyan 22 and Mingxian 169 was less than 20% after seed treatment with *G. lucidum* polysaccharides at doses of 4 g/100 kg and 8 g/100 kg, although it exceeded 15% in Xinong 873 and Sumai 3. The disease control efficacy against wheat sharp eyespot in Xiaoyan 22, Sumai 3, Mingxian 169, and Xinong 873 rose by more than 44% after a 7-day treatment with *G. lucidum* polysaccharides at a seed dose of 8 g/100 kg compared to the treatment with a seed dose of 4 g/100 kg.

### 3.5. Effect of G. lucidum Polysaccharide Seed Treatment on Chlorophyll a and Chlorophyll b Content in Wheat Leaves

#### 3.5.1. Effects of Fusarium Head Blight on Chlorophyll a and Chlorophyll b Content in Wheat Leaves

**Figure 2:**
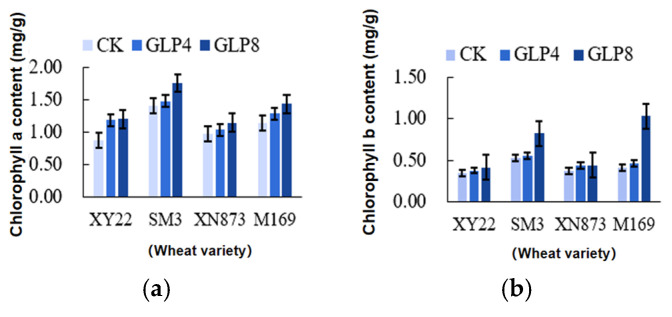
Effects of *G. lucidum* polysaccharide seed dressing on chlorophyll a and chlorophyll b contents in wheat leaves with *Fusarium* head blight. (**a**) Chlorophyll a content; (**b**) Chlorophyll b content. CK, blank control without GLP treatment; GLP4 and GLP8 represent GLP seed-dressing dosages of 4 g per 100 kg seeds and 8 g per 100 kg seeds, respectively. XY22 = Xiaoyan 22, SM3 = Sumai 3, XN873 = Xinong 873, M169 = Mingxian 169. Vertical error bars represent standard error of the mean.

After applying 8 g/100 kg *G. lucidum* polysaccharides to seeds for 14 days, Xiaoyan 22 had the highest chlorophyll a content (1.34 mg/g), which was 6% higher than the control group; Mingxian 169 had the highest chlorophyll b content (0.49 mg/g), which was 48% higher than the control group. At 1.10 and 0.36 mg/g, respectively—increases of 3% and 7% over the control group—Xinong 873 showed the lowest levels of chlorophyll a and b. Xiaoyan 22 showed the lowest chlorophyll a and b contents at 1.33 and 0.44 mg/g, respectively, representing increases of 19% and 17% over the control group, while Sumai 3 showed the highest chlorophyll a and b contents at 1.68 and 0.62 mg/g, respectively, after 21 days of seed treatment with 8 g/100 kg *G. lucidum* polysaccharides (Figure 2).

#### 3.5.2. Effects of *G. lucidum* Polysaccharide Seed Dressing on Chlorophyll a and Chlorophyll b Contents in Leaves of Wheat Infected with Wheat Sharp Eyespot

**Figure 3:**
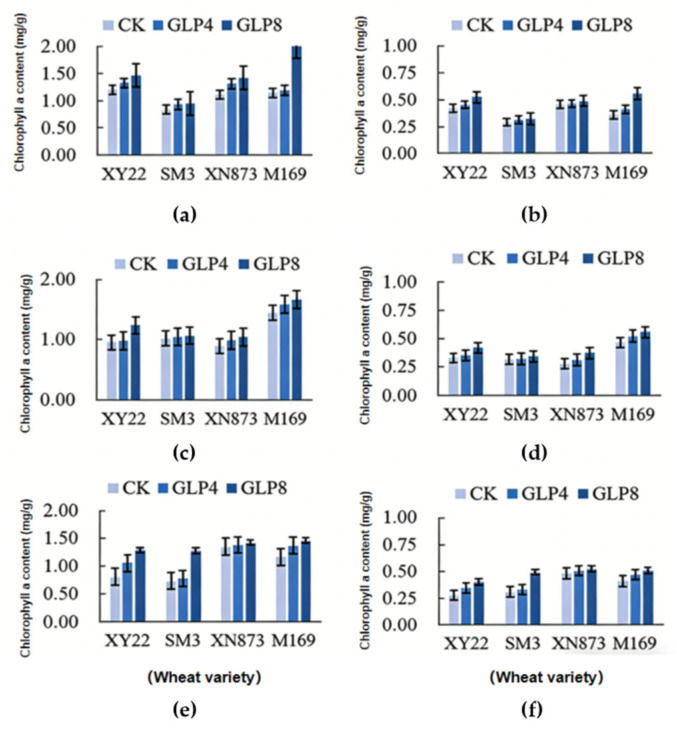
Effect of *G. lucidum* polysaccharide seed dressing on chlorophyll a and chlorophyll b contents in wheat leaves with wheat sharp eyespot. (**a**) Chlorophyll a content at 7 d after pathogen inoculation; (**b**) Chlorophyll b content at 7 d after pathogen inoculation; (**c**) Chlorophyll a content at 14 d after pathogen inoculation; (**d**) Chlorophyll b content at 14 d after pathogen inoculation; (**e**) Chlorophyll a content at 21 d after pathogen inoculation; (**f**) Chlorophyll b content at 21 d after pathogen inoculation. CK, blank control without GLP treatment; GLP4 and GLP8 represent GLP seed-dressing dosages of 4 g per 100 kg seeds and 8 g per 100 kg seeds, respectively. XY22 = Xiaoyan 22, SM3 = Sumai 3, XN873 = Xinong 873, M169 = Mingxian 169. Vertical error bars represent standard error of the mean.

Following a 14-day treatment of seeds with *G. lucidum* polysaccharides at 8 g/100 kg, Mingxian 169 showed the highest levels of chlorophyll a and b (1.66 and 0.55 mg/g, respectively), indicating increases of 16% and 20% over the control group; Xinong 873 showed the lowest levels of chlorophyll a (1.04 mg/g, representing a 16% increase over the control group; and Sumai 3 showed the lowest levels of chlorophyll b (0.34 mg/g), indicating a 7% increase over the control group. After seven days of seed treatment with 8 g/100 kg *G. lucidum* polysaccharides, Mingxian 169 showed the highest levels of chlorophyll a and b at 1.99 and 0.56 mg/g, respectively, indicating increases of 74% and 56% over the control group; Sumai 3 showed the lowest levels at 0.95 and 0.32 mg/g, respectively, indicating increases of 14% and 10% over the control group. After a 21-day seed treatment with 8 g/100 kg *G. lucidum* polysaccharides, Mingxian 169 had the highest chlorophyll a content (1.45 mg/g), which was 25% higher than the control group; Xinong 873 had the highest chlorophyll b content (0.52 mg/g), which was 8% higher than the control group. Sumai 3 had the lowest chlorophyll a content (1.27 mg/g), which was 72% higher than the control group; Xiaoyan 22 had the lowest chlorophyll b content (0.41 mg/g), which was 46% higher than the control group (Figure 3).

### 3.6. G. lucidum Polysaccharide Seed Dressing’s Impact on Wheat’s Malondialdehyde Concentration

#### 3.6.1. Effects of *G. lucidum* Polysaccharide Seed Dressing on MDA Content in Wheat Leaves Infected with Fusarium Head Blight

**Figure 4:**
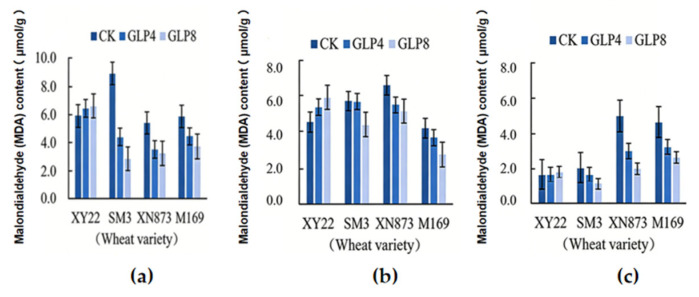
Effect of *G. lucidum* polysaccharide seed dressing on MDA content of wheat with *Fusarium* head blight. (**a**) MDA content at 7 days post inoculation; (**b**) MDA content at 14 days post inoculation; (**c**) MDA content at 21 days post inoculation. CK = blank control; GLP4 = 4 g GLP per 100 kg seeds, GLP8 = 8 g GLP per 100 kg seeds. XY22, SM3, XN873, M169 are abbreviations for Xiaoyan 22, Sumai 3, Xinong 873 and Mingxian 169. Vertical error bars indicate standard error of the mean.

After applying 8 g/100 kg of *G. lucidum* polysaccharides to the seeds for 7 days, Sumai 3′s MDA level dropped to at least 2.84 μmol/g, a 68% decrease from the control group. On the other hand, Xiao Yan 22 had a peak MDA content of 6.59 μmol/g, which was 11% higher than the control. After being treated with *G. lucidum* polysaccharides at 8 g/100 kg for 14 days, Mingxian 169 had the lowest MDA content (2.76 μmol/g), which was 34% lower than the control group; Xiaoyan 22 had the highest MDA content (5.89 μmol/g), which was 23% higher than the control. After 21 days of seed treatment with 8 g/100 kg *G. lucidum* polysaccharides, Mingxian 169 had the highest MDA content (2.62 μmol/g), which was 44% lower than the control group; Sumai 3 had the lowest MDA content (1.06 μmol/g), which was 48% lower than the control group (Figure 4).

#### 3.6.2. Effects of *G. lucidum* Polysaccharide Seed Dressing on MDA Content in Wheat Leaves Infected with Sharp Eyespot

**Figure 5:**
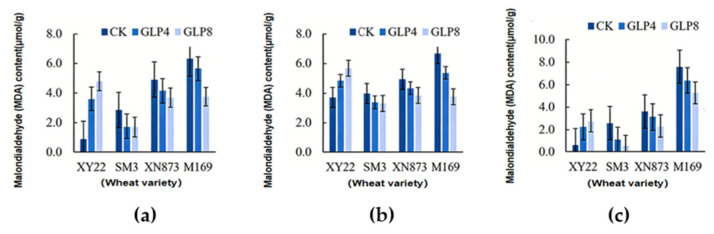
Effect of *G. lucidum* polysaccharide seed dressing on MDA content of wheat with wheat sharp eyespot. (**a**) MDA content at 7 d after pathogen inoculation; (**b**) MDA content at 14 d after pathogen inoculation; (**c**) MDA content at 21 d after pathogen inoculation. CK, blank control without GLP treatment; GLP4 and GLP8 represent GLP seed-dressing dosages of 4 g per 100 kg seeds and 8 g per 100 kg seeds, respectively. XY22 = Xiaoyan 22, SM3 = Sumai 3, XN873 = Xinong 873, M169 = Mingxian 169. Vertical error bars represent standard error of the mean.

At 7 days post-seed dressing with 8 g/100 kg GLP, Sumai 3 exhibited the lowest MDA content of 1.70 μmol/g, which was 41% lower than the control group. Xiaoyan 22 had the highest MDA content at 4.80 μmol/g, representing an 82% increase relative to the control. At 14 days after the same GLP seed dressing treatment, Sumai 3 still showed the minimum MDA level of 3.30 μmol/g, a 17% reduction compared with the control. The maximum MDA content of 5.70 μmol/g was recorded in Xiaoyan 22, which was 45% higher than that of the control. At 21 days post-treatment, Sumai 3 maintained the lowest MDA content of 0.54 μmol/g, decreasing by 79% relative to the control. Mingxian 169 had the highest MDA concentration of 5.26 μmol/g, which was 31% lower than the control (Figure 5).

## 4. Discussion

This study demonstrates that seed coating with *G. lucidum* polysaccharide (GLP) exerts dual beneficial effects on wheat: it suppresses two major fungal diseases, FHB and wheat sharp eyespot, while improving multiple physiological traits. Disease control data confirm that GLP seed treatment exerts definite control efficacy against both pathogens, with significant cultivar-dependent variations in control efficiency. Specifically, the moderately resistant cultivar Sumai 3 exhibited the highest control efficacy against FHB, exceeding 50%. The moderately resistant cultivar Xinong 873 showed outstanding control against wheat sharp eyespot, with a control efficacy reaching 100% at 7 days post-inoculation (dpi), indicating that its strong inherent resistance acts synergistically with GLP seed coating for disease management. However, GLP showed relatively poor control efficacy against wheat sharp eyespot in some wheat cultivars. Under the treatment of 8 g GLP per 100 kg seeds at 7 dpi, the control efficacies against sharp eyespot in Xiaoyan 22, Sumai 3 and Mingxian 169 were all below 30%. It is speculated that severe disease infection masks the disease-preventing potential of GLP. Previous studies on pepper seedling cultivation have also confirmed that the efficacy of plant immune elicitors is closely related to the inherent resistance level of crop cultivars, and highly resistant cultivars respond better to elicitor treatments [42]. The most prominent increase in seed germination rate was observed in the moderately susceptible cultivar Mingxian 169, with a 19.5% elevation under the 8 g/100 kg treatment, which is consistent with previous reports that GLP promotes seed germination in crops [24,25]. Differential responses were observed among cultivars: the moderately resistant Sumai 3 and moderately susceptible Mingxian 169 showed the most remarkable growth promotion, indicating that the effects of GLP are cultivar-dependent. This finding aligns with reports on the bioactivities of fungal polysaccharides such as lentinan and chitosan oligosaccharide in crops [43,44,45].

Accumulating evidence has verified that fungal polysaccharides can elevate chlorophyll content in plants, thereby promoting plant growth and development [46,47]. Zeng et al. reported that delaying chlorophyll degradation in plant leaves can inhibit the occurrence and progression of plant diseases [48]. Higher leaf chlorophyll content confers stronger photosynthesis and a more sufficient energy supply, which in turn enhances plant disease resistance. The results of this study are consistent with the above conclusions: GLP treatment significantly increased chlorophyll a and chlorophyll b contents in all tested cultivars, with an overall increase of over 5% and a maximum increase of 74%, indicating that GLP seed coating effectively alleviates photosynthetic pigment degradation caused by disease stress. Maintenance of chlorophyll content supports normal photosynthesis and provides material and energy bases for plants to cope with pathogen infection, which may be one of the key physiological mechanisms underlying GLP-enhanced wheat disease resistance.

MDA is a product of lipid peroxidation, and its content reflects the degree of damage to the cell membrane system [49,50]. In this study, GLP treatment significantly reduced MDA content in Sumai 3 (with a reduction rate up to 68%), as well as in moderately susceptible cultivars Mingxian 169 and Xinong 873, suggesting that GLP seed coating mitigates disease-induced lipid peroxidation. This is consistent with reports that polysaccharide elicitors reduce MDA accumulation in plants under stress [51,52,53]. However, Xiaoyan 22 exhibited elevated MDA content under both disease stresses, contrary to the trends observed in other cultivars; the underlying mechanism requires further investigation. These cultivar-specific differences in MDA responses suggest that the alleviating effect of GLP on lipid peroxidation varies by cultivar, and its mechanism may be associated with the inherent disease tolerance characteristics of each cultivar.

Collectively, these results indicate that GLP seed coating exerts multiple effects on wheat, including growth promotion, photosynthetic pigment maintenance, lipid peroxidation alleviation, and disease suppression. In cultivars such as Sumai 3, maintained chlorophyll content and reduced MDA levels coincided with higher disease control efficacy, suggesting that improvements in these physiological indicators serve as important physiological foundations for GLP-enhanced wheat disease resistance. From a practical application perspective, the differential responses among cultivars indicate that cultivar characteristics should be taken into account when applying GLP. This study provides experimental evidence for GLP as a bio-based seed coating agent with both growth-promoting and disease-preventing functions, and offers a reference for its rational application in integrated wheat disease management.

## 5. Conclusions

Seed soaking with GLP improves the germination rate and seedling growth of wheat varieties Xiaoyan 22, Sumai 3 and Mingxian 169, while no significant growth-promoting effect is observed on Xinong 873. This treatment alleviates plant damage caused by FHB and wheat sharp eyespot, increases the contents of leaf chlorophyll a and chlorophyll b, and such effects are strengthened with the elevation of GLP concentration. Distinct genotypic differences exist in the physiological responses of tested wheat varieties; the MDA levels in Sumai 3, Mingxian 169, and Xinong 873 are lower than those in Xiaoyan 22. The present study verifies that GLP seed soaking positively regulates growth and disease resistance-related physiological indicators in most tested wheat varieties.

## Figures and Tables

**Table 1:** Effect of *G. lucidum* polysaccharide seed dressing on wheat seed germination rate.

Wheat Variety	Treatment *	Germination Percentage (%)
Xiaoyan 22	CK	84.78 ± 2.72
	GLP4	86.00 ± 3.50
	GLP8	88.00 ± 1.29
Sumai 3	CK	94.00 ± 3.10
	GLP4	97.78 ± 3.70
	GLP8	100 ± 0.00
Mingxian 169	CK	80.43 ± 0.83
	GLP4	95.56 ± 0.73
	GLP8	96.15 ± 1.39
Xinong 873	CK	86.67 ± 1.24
	GLP4	88.46 ± 2.91
	GLP8	88.89 ± 0.35

* CK, blank control without GLP treatment; GLP4 and GLP8 represent GLP seed-dressing dosages of 4 g per 100 kg of seeds and 8 g per 100 kg of seeds, respectively. Data on the control effect are presented as mean ± standard error.

**Table 2:** Effects of *G. lucidum* polysaccharide seed dressing on the growth of wheat seedlings with *Fusarium* head blight.

Wheat Variety	Treatment *	Plant Height/cm		
		7 d	14 d	21 d
Xiaoyan 22	CK	12.37 ± 3.21 b	16.22 ± 3.56 b	18.25 ± 2.97 b
	GLP4	14.96 ± 3.48 a	18.45 ± 3.91 ab	20.73 ± 4.35 ab
	GLP8	17.43 ± 2.55 a	21.02 ± 1.61 a	22.53 ± 1.67 a
Sumai 3	CK	17.54 ± 4.04 b	23.99 ± 4.71 b	32.21 ± 3.15 c
	GLP4	20.36 ± 2.80 a	26.13 ± 3.77 ab	34.28 ± 3.39 b
	GLP8	20.83 ± 1.92 a	27.21 ± 2.74 a	37.09 ± 2.87 a
Mingxian 169	CK	12.90 ± 3.42 b	15.58 ± 4.01 b	20.41 ± 4.12 b
	GLP4	15.04 ± 2.55 a	18.27 ± 1.48 a	23.12 ± 4.30 ab
	GLP8	15.63 ± 1.50 a	18.70 ± 2.99 a	23.55 ± 1.70 a
Xinong 873	CK	10.87 ± 1.77 b	14.29 ± 1.77 b	18.28 ± 2.24 b
	GLP4	11.50 ± 2.03 b	14.41 ± 1.53 b	18.51 ± 2.04 b
	GLP8	12.63 ± 1.22 a	15.91 ± 1.22 a	20.46 ± 1.21 a

* CK, blank control without GLP treatment; GLP4 and GLP8 represent GLP seed-dressing dosages of 4 g per 100 kg of seeds and 8 g per 100 kg of seeds, respectively. 7 d, 14 d, and 21 d indicate the days after seedling emergence. Different lowercase letters within the same column denote significant differences at *p* < 0.05.

**Table 3:** Effects of *G. lucidum* polysaccharide seed dressing on the growth of wheat seedlings with wheat sharp eyespot.

Wheat Variety	Treatment *	Plant Height/cm		
		7 d	14 d	21 d
Xiaoyan 22	CK	8.78 ± 5.66 b	14.84 ± 5.08 b	18.06 ± 2.89 b
	GLP4	12.29 ± 4.54 ab	14.96 ± 5.64 b	19.38 ± 3.16 ab
	GLP8	16.29 ± 4.13 a	20.41 ± 3.56 a	21.32 ± 2.26 a
Sumai 3	CK	19.70 ± 2.86 b	22.42 ± 3.66 b	27.01 ± 4.03 b
	GLP4	21.68 ± 3.05 a	24.61 ± 3.02 a	29.84 ± 6.17 ab
	GLP8	21.98 ± 1.61 a	25.94 ± 2.38 a	32.38 ± 5.11 a
Mingxian 169	CK	7.40 ± 2.32 b	11.91 ± 2.13 b	13.43 ± 2.97 c
	GLP4	12.62 ± 4.03 a	15.18 ± 2.45 a	16.27 ± 4.83 b
	GLP8	12.89 ± 2.40 a	15.24 ± 4.33 a	20.20 ± 2.38 a
Xinong 873	CK	9.05 ± 2.72 c	12.20 ± 2.85 b	17.02 ± 2.90 b
	GLP4	10.71 ± 1.46 b	14.41 ± 1.59 a	18.36 ± 2.07 ab
	GLP8	12.43 ± 1.86 a	14.99 ± 1.68 a	18.93 ± 1.70 a

* CK indicates blank control without GLP treatment; GLP4 and GLP8 represent GLP seed-dressing dosages of 4 g per 100 kg of seeds and 8 g per 100 kg of seeds, respectively. 7 d, 14 d and 21 d refer to days after seedling emergence. Different lowercase letters within the same column indicate significant differences at *p* < 0.05.

**Table 4:** Control effect of *G. lucidum* polysaccharide seed dressing on wheat *Fusarium* head blight.

Wheat Variety	Treatment *	7 d		14 d		21 d	
		Disease Index	Control Effect (%)	Disease Index	Control Effect (%)	Disease Index	Control Effect (%)
Xiaoyan 22	CK	52.85	/	59.43	/	63.93	/
	GLP4	45.00	14.9 ± 0.09	57.80	2.7 ± 0.02	60.79	4.9 ± 0.03
	GLP8	35.00	33.8 ± 0.12	40.00	32.7 ± 0.18	44.00	31.2 ± 0.02
Sumai 3	CK	48.50	/	50.00	/	58.10	/
	GLP4	24.00	50.5 ± 0.28	25.22	49.6 ± 0.16	28.60	50.8 ± 0.11
	GLP8	20.00	58.8 ± 0.10	24.29	51.4 ± 0.20	29.00	50.1 ± 0.07
Mingxian 169	CK	45.00	/	63.00	/	85.00	/
	GLP4	36.70	17.0 ± 0.01	53.00	15.9 ± 0.13	75.00	11.8 ± 0.06
	GLP8	28.30	37.1 ± 0.11	36.00	42.9 ± 0.02	55.00	35.3 ± 0.29
Xinong 873	CK	28.57	/	33.30	/	40.00	/
	GLP4	21.54	24.6 ± 0.17	23.00	30.9 ± 0.12	28.60	34.1 ± 0.16
	GLP8	19.05	33.3 ± 0.02	20.00	39.9 ± 0.26	20.00	50.0 ± 0.03

* CK, blank control without GLP treatment; GLP4 and GLP8 represent GLP seed-dressing dosages of 4 g per 100 kg of seeds and 8 g per 100 kg of seeds, respectively. 7 d, 14 d and 21 d denote days after pathogen inoculation. Slash (/) indicates no control effect value for the blank control group. Data of control effect are presented as mean ± standard error.

**Table 5:** Control effect of *G. lucidum* polysaccharide seed dressing on wheat sharp eyespot.

Wheat Variety	Treatment *	7 d		14 d		21 d	
		Disease Index	Control Effect (%)	Disease Index	Control Effect (%)	Disease Index	Control Effect (%)
Xiaoyan 22	CK	72.85	/	74.21	/	79.59	/
	GLP4	65.70	9.8 ± 0.02	68.15	8.2 ± 0.04	75.72	4.9 ± 0.00
	GLP8	65.00	12.8 ± 0.09	67.15	9.5 ± 0.03	73.10	8.2 ± 0.04
Sumai 3	CK	35.72	/	45.72	/	47.10	/
	GLP4	28.58	20.0 ± 0.09	38.26	16.3 ± 0.09	39.10	17.0 ± 0.03
	GLP8	26.79	25.0 ± 0.02	26.99	41.0 ± 0.03	28.60	39.3 ± 0.08
Mingxian 169	CK	54.70	/	56.20	/	75.00	/
	GLP4	52.85	3.4 ± 0.01	55.65	1.0 ± 0.01	61.65	17.8 ± 0.11
	GLP8	52.00	4.9 ± 0.04	52.86	5.9 ± 0.00	53.50	28.7 ± 0.09
Xinong 873	CK	50.00	/	53.57	/	57.50	/
	GLP4	28.57	42.9 ± 0.07	38.10	28.9 ± 0.00	40.00	30.4 ± 0.09
	GLP8	0.14 ± 0.01	100.0 ± 0.00	17.86	66.7 ± 0.02	28.60	50.3 ± 0.01

* CK, blank control without GLP treatment; GLP4 and GLP8 represent GLP seed-dressing dosages of 4 g per 100 kg of seeds and 8 g per 100 kg of seeds, respectively. 7 d, 14 d and 21 d denote days after pathogen inoculation. Slash (/) indicates no control effect value for the blank control group. Data of control effect are presented as mean ± standard error.

## Data Availability

The data supporting the findings of this study are available from the corresponding author upon reasonable request.
